# Bis(2,6-dimethyl­phenyl isocyanide-κ*C*)gold(I) tetra­fluorido­borate

**DOI:** 10.1107/S1600536808027116

**Published:** 2008-09-06

**Authors:** Timi P. Singa, Antonio G. DiPasquale, Arnold L. Rheingold, Clifford P. Kubiak

**Affiliations:** a University of California in San Diego, Department of Chemistry and Biochemistry, 9500 Gilman Drive, La Jolla, California 92093-0358, USA

## Abstract

In the title compound, [Au(C_9_H_9_N)_2_]BF_4_, the Au^I^ cation adopts an almost linear AuC_2_ geometry. The cation is bowed due to crystal packing effects, and the dihedral angle between the xylyl rings is 52.3 (7)°.

## Related literature

For related literature, see: Balch & Parks (1973 ▶, 1974 ▶); Bonati & Minghetti (1973 ▶); Schmidbaur *et al.* (1997 ▶, 2002 ▶).
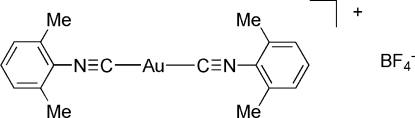


## Experimental

### 

#### Crystal data

[Au(C_9_H_9_N)_2_]BF_4_
*M* *_r_* = 546.15
Monoclinic, 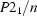

*a* = 13.1930 (15) Å
*b* = 10.7840 (13) Å
*c* = 13.6260 (15) Å
β = 105.034 (2)°
*V* = 1872.3 (4) Å^3^
*Z* = 4
Mo *K*α radiation
μ = 7.90 mm^−1^
*T* = 208 (2) K
0.20 × 0.12 × 0.07 mm

#### Data collection

Bruker SMART CCD diffractometer
Absorption correction: multi-scan (*SADABS*; Bruker, 2003 ▶) *T*_min_ = 0.301, *T*_max_ = 0.608 (expected range = 0.285–0.575)
18352 measured reflections
3302 independent reflections
3011 reflections with *I* > 2σ(*I*)
*R*_int_ = 0.028

#### Refinement

*R*[*F* ^2^ > 2σ(*F* ^2^)] = 0.060
*wR*(*F* ^2^) = 0.174
*S* = 1.10
3302 reflections
241 parameters
H-atom parameters constrained
Δρ_max_ = 2.19 e Å^−3^
Δρ_min_ = −1.01 e Å^−3^

### 

Data collection: *SMART* (Bruker, 2003 ▶); cell refinement: *SAINT* (Bruker, 2006 ▶); data reduction: *SAINT*; program(s) used to solve structure: *SIR2004* (Burla *et al.*, 2005 ▶); program(s) used to refine structure: *SHELXL97* (Sheldrick, 2008 ▶); molecular graphics: *ORTEP-32* (Farrugia, 1997 ▶); software used to prepare material for publication: *WinGX* (Farrugia, 1999 ▶).

## Supplementary Material

Crystal structure: contains datablocks I, global. DOI: 10.1107/S1600536808027116/hb2785sup1.cif

Structure factors: contains datablocks I. DOI: 10.1107/S1600536808027116/hb2785Isup2.hkl

Additional supplementary materials:  crystallographic information; 3D view; checkCIF report

## Figures and Tables

**Table 1:** Selected bond lengths (Å)

Au1—C1	2.068 (9)
Au1—C10	2.035 (8)

**Fig. 1.**
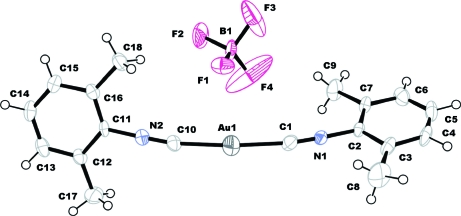
The molecular structure of (I) showing 50% displacement ellipsoids for the non-hydrogen atoms.

## supplementary crystallographic information

### Comment

Gold bis-isocyanide complexes of the type (RNC)_2_Au^I^ have been studied as precursors to the related carbene complexes of gold (Balch & Parks, 1973; Bonati & Minghetti, 1973; Balch & Parks, 1974). These early examples were characterized by NMR, IR, elemental analysis, and conductivity studies. More recently bis(*tert*-butyl isocyanide)gold(I) (Schmidbaur *et al.*, 2002) and various aromatic isocyanide complexes of gold (Schmidbaur *et al.*, 1997) have been studied by *x*-ray crystallography. Here, the title compound, (I), has been structurally characterised (Fig. 1).

The structure of the cation in (I) is nearly linear, with the C—Au—C bond angle at 171.2 (7)°. The bow in the structure is due to the crystal packing, and has been observed in bis(isonitrile) gold cations containing methyl, *tert*-butyl, phenyl, or mesistyl groups attached to the isonitrile groups (Schmidbaur *et al.*, 1997). The Au—C distances in (I) are given in Table 1. Both these bond lengths are slightly longer than the gold-carbon bond distances given for the phenyl and mesityl analogues of (I) (Schmidbaur *et al.*, 1997). The bond length between C1—N1 is 1.124 (11)Å, and the length between C10 and N2 is 1.154 (11)Å. These length are, again, slightly longer than those reported for the phenyl and mesityl analogues, but the difference between the C1—N1 bond and the C10—N2 bond in (I) is also present in the isocyanide complexes studied (Schmidbaur*et al.*, 1997, Schmidbaur *et al.*, 2002). The slightly longer bond lengths in (I) could be due to decreased electron density in the C1—N1 and C10—N2 bonds. That electron density would be shifted toward the xylyl ring through resonance stabilization. The xylyl groups of the cation in (I) define planes that are orientated at an angle of 52.3 (7)°.

### Experimental

HAuCl_4_.H_2_O (Acros Organics, 0.5 g) was dissolved in ethyl acetate resulting in a pale yellow solution. The dimethyl phenyl isocyanide (Aldrich, 0.5 g) was added following the complete dissolution of the HAuCl_4_.H_2_O. Upon addition, the solution immediately became cloudy and brown in color. Methanol was added drop-wise until the precipitate was dissolved. AgBF_4_ (Aldrich, 0.25 g) was added resulting in the product formation and precipitation of AgCl. The solvent was removed *in vacuo* and reddish-brown crystals were obtained (90% yield, crude product). The solid was suspended in diethyl ether and allowed to stir for an hour. The solid was filtered, then dissolved in dichloromethane and subsequent recrystallisations yielded pure white powder (12% yield). Colourless blocks of (I) were obtained through the slow diffusion of diethyl ether into a dichloromethane solution. IR (KBr) \vCN 2224 cm^-1^; ^1^H NMR, acetone-*d_6_*, CH_3_ singlet 2.51ppm, Ar—H mult 7.34ppm; Molecular ion peak, ESI positive mode 459.06 *^m^*/*_z_*

### Refinement

The H atoms were geometrically placed (C—H = 0.94-0.97Å) and refined as riding with U_iso_(H) = 1.2U_eq_(C) or 1.5U_eq_(methyl C).

### Crystal data

**Table:**

[Au(C_9_H_9_N)_2_]BF_4_	*F*(000) = 1040
*M**_r_* = 546.15	*D*_x_ = 1.938 Mg m^−^^3^
Monoclinic, *P*2_1_/*n*	Mo *K*α radiation, λ = 0.71073 Å
Hall symbol: -P 2yn	Cell parameters from 6164 reflections
*a* = 13.1930 (15) Å	θ = 2.5–28.2°
*b* = 10.7840 (13) Å	µ = 7.90 mm^−^^1^
*c* = 13.6260 (15) Å	*T* = 208 K
β = 105.034 (2)°	Block, colorless
*V* = 1872.3 (4) Å^3^	0.20 × 0.12 × 0.07 mm
*Z* = 4	

### Data collection

**Table:**

Bruker SMART CCD diffractometer	3302 independent reflections
Radiation source: fine-focus sealed tube	3011 reflections with *I* > 2σ(*I*)
graphite	*R*_int_ = 0.028
ω scans	θ_max_ = 25.0°, θ_min_ = 2.4°
Absorption correction: multi-scan (SADABS; Bruker, 2003)	*h* = −15→15
*T*_min_ = 0.301, *T*_max_ = 0.608	*k* = −12→12
18352 measured reflections	*l* = −16→16

### Refinement

**Table:**

Refinement on *F*^2^	Primary atom site location: structure-invariant direct methods
Least-squares matrix: full	Secondary atom site location: difference Fourier map
*R*[*F*^2^ > 2σ(*F*^2^)] = 0.060	Hydrogen site location: inferred from neighbouring sites
*wR*(*F*^2^) = 0.174	H-atom parameters constrained
*S* = 1.10	*w* = 1/[σ^2^(*F*_o_^2^) + (0.1125*P*)^2^ + 10.5769*P*] where *P* = (*F*_o_^2^ + 2*F*_c_^2^)/3
3302 reflections	(Δ/σ)_max_ = 0.001
241 parameters	Δρ_max_ = 2.19 e Å^−^^3^
0 restraints	Δρ_min_ = −1.02 e Å^−^^3^

### Special details

**Table:**

Geometry. All e.s.d.'s (except the e.s.d. in the dihedral angle between two l.s. planes) are estimated using the full covariance matrix. The cell e.s.d.'s are taken into account individually in the estimation of e.s.d.'s in distances, angles and torsion angles; correlations between e.s.d.'s in cell parameters are only used when they are defined by crystal symmetry. An approximate (isotropic) treatment of cell e.s.d.'s is used for estimating e.s.d.'s involving l.s. planes.
Refinement. Refinement of *F*^2^ against ALL reflections. The weighted *R*-factor *wR* and goodness of fit *S* are based on *F*^2^, conventional *R*-factors *R* are based on *F*, with *F* set to zero for negative *F*^2^. The threshold expression of *F*^2^ > σ(*F*^2^) is used only for calculating *R*-factors(gt) *etc*. and is not relevant to the choice of reflections for refinement. *R*-factors based on *F*^2^ are statistically about twice as large as those based on *F*, and *R*- factors based on ALL data will be even larger.

### Fractional atomic coordinates and isotropic or equivalent isotropic displacement parameters (Å^2^)

**Table:**

	*x*	*y*	*z*	*U*_iso_*/*U*_eq_	
C1	0.9280 (7)	0.0807 (7)	0.3856 (6)	0.0336 (17)	
C2	0.8668 (5)	0.1409 (6)	0.5405 (5)	0.0222 (13)	
C3	0.7920 (6)	0.0703 (7)	0.5708 (7)	0.0355 (18)	
C4	0.7599 (6)	0.1149 (9)	0.6546 (6)	0.043 (2)	
H4	0.7089	0.0706	0.6773	0.051*	
C5	0.8002 (6)	0.2200 (9)	0.7038 (6)	0.044 (2)	
H5	0.7760	0.2480	0.7590	0.053*	
C6	0.8765 (6)	0.2866 (8)	0.6739 (6)	0.0399 (18)	
H6	0.9052	0.3579	0.7104	0.048*	
C7	0.9112 (5)	0.2494 (7)	0.5905 (5)	0.0285 (14)	
C8	0.7478 (9)	−0.0460 (10)	0.5127 (11)	0.067 (3)	
H8A	0.7995	−0.1119	0.5291	0.101*	
H8B	0.7308	−0.0293	0.4403	0.101*	
H8C	0.6848	−0.0712	0.5315	0.101*	
C9	0.9954 (7)	0.3209 (9)	0.5590 (7)	0.049 (2)	
H9A	1.0151	0.3926	0.6028	0.073*	
H9B	0.9692	0.3481	0.4891	0.073*	
H9C	1.0562	0.2681	0.5647	0.073*	
C10	1.0572 (6)	0.0646 (6)	0.1430 (6)	0.0292 (16)	
C11	1.1341 (5)	0.1056 (7)	−0.0065 (5)	0.0229 (13)	
C12	1.2073 (5)	0.0216 (7)	−0.0291 (6)	0.0288 (15)	
C13	1.2437 (6)	0.0482 (8)	−0.1132 (7)	0.0386 (19)	
H13	1.2922	−0.0052	−0.1311	0.046*	
C14	1.2092 (6)	0.1533 (8)	−0.1714 (6)	0.0375 (17)	
H14	1.2359	0.1710	−0.2274	0.045*	
C15	1.1366 (6)	0.2318 (7)	−0.1482 (6)	0.0335 (16)	
H15	1.1132	0.3012	−0.1896	0.040*	
C16	1.0972 (5)	0.2104 (6)	−0.0645 (5)	0.0268 (14)	
C17	1.2444 (8)	−0.0907 (9)	0.0365 (8)	0.050 (2)	
H17A	1.1842	−0.1371	0.0449	0.075*	
H17B	1.2867	−0.0642	0.1025	0.075*	
H17C	1.2863	−0.1430	0.0043	0.075*	
C18	1.0179 (6)	0.2961 (8)	−0.0383 (6)	0.0393 (17)	
H18A	1.0034	0.3644	−0.0863	0.059*	
H18B	1.0456	0.3283	0.0299	0.059*	
H18C	0.9535	0.2509	−0.0416	0.059*	
B1	0.9656 (9)	0.3821 (9)	0.2329 (7)	0.044 (2)	
N1	0.9001 (5)	0.1048 (6)	0.4545 (5)	0.0264 (12)	
N2	1.0945 (5)	0.0817 (6)	0.0764 (5)	0.0270 (13)	
F1	0.9014 (4)	0.2940 (4)	0.1733 (4)	0.0481 (12)	
F2	1.0249 (6)	0.4422 (6)	0.1808 (6)	0.075 (2)	
F3	0.9000 (8)	0.4761 (9)	0.2512 (10)	0.128 (4)	
F4	1.0161 (12)	0.3331 (11)	0.3187 (7)	0.190 (8)	
Au1	0.98971 (3)	0.06293 (4)	0.26147 (3)	0.0551 (2)	

### Atomic displacement parameters (Å^2^)

**Table:**

	*U*^11^	*U*^22^	*U*^33^	*U*^12^	*U*^13^	*U*^23^
C1	0.041 (4)	0.031 (4)	0.028 (4)	−0.002 (3)	0.008 (3)	0.004 (3)
C2	0.018 (3)	0.032 (3)	0.016 (3)	0.000 (2)	0.004 (2)	0.003 (2)
C3	0.028 (4)	0.045 (5)	0.035 (4)	−0.007 (3)	0.011 (3)	0.012 (3)
C4	0.019 (3)	0.074 (6)	0.040 (4)	0.009 (4)	0.017 (3)	0.028 (5)
C5	0.036 (4)	0.075 (6)	0.025 (4)	0.023 (4)	0.015 (3)	0.016 (4)
C6	0.044 (4)	0.048 (5)	0.026 (4)	0.006 (4)	0.007 (3)	−0.006 (3)
C7	0.026 (3)	0.038 (4)	0.023 (3)	−0.006 (3)	0.010 (3)	0.002 (3)
C8	0.063 (7)	0.061 (6)	0.083 (8)	−0.039 (5)	0.029 (6)	−0.014 (6)
C9	0.046 (5)	0.052 (5)	0.053 (5)	−0.030 (4)	0.024 (4)	−0.012 (4)
C10	0.022 (3)	0.035 (4)	0.030 (4)	0.005 (3)	0.006 (3)	0.000 (3)
C11	0.018 (3)	0.035 (3)	0.019 (3)	−0.004 (3)	0.010 (2)	−0.006 (3)
C12	0.020 (3)	0.036 (4)	0.031 (4)	0.006 (3)	0.007 (3)	−0.001 (3)
C13	0.028 (4)	0.053 (5)	0.040 (5)	0.004 (3)	0.017 (3)	−0.008 (4)
C14	0.032 (4)	0.057 (5)	0.028 (4)	−0.004 (3)	0.016 (3)	−0.001 (3)
C15	0.035 (4)	0.041 (4)	0.026 (3)	−0.005 (3)	0.010 (3)	0.003 (3)
C16	0.021 (3)	0.032 (3)	0.027 (3)	−0.001 (3)	0.006 (3)	−0.006 (3)
C17	0.045 (5)	0.050 (5)	0.058 (6)	0.020 (4)	0.020 (4)	0.016 (4)
C18	0.039 (4)	0.044 (4)	0.038 (4)	0.010 (3)	0.015 (3)	−0.003 (3)
B1	0.071 (6)	0.039 (5)	0.034 (5)	−0.022 (5)	0.037 (5)	−0.011 (4)
N1	0.031 (3)	0.026 (3)	0.023 (3)	0.000 (2)	0.009 (2)	0.002 (2)
N2	0.022 (3)	0.033 (3)	0.025 (3)	−0.001 (2)	0.004 (3)	−0.003 (2)
F1	0.052 (3)	0.035 (2)	0.047 (3)	0.000 (2)	−0.006 (2)	−0.006 (2)
F2	0.083 (5)	0.083 (5)	0.083 (5)	−0.011 (3)	0.064 (4)	0.005 (3)
F3	0.130 (7)	0.099 (6)	0.200 (11)	−0.054 (6)	0.125 (8)	−0.093 (7)
F4	0.266 (14)	0.155 (9)	0.072 (6)	−0.145 (10)	−0.096 (8)	0.055 (6)
Au1	0.0669 (3)	0.0573 (3)	0.0455 (3)	0.00923 (16)	0.0225 (2)	0.00368 (15)

### Geometric parameters (Å, °)

**Table:**

Au1—C1	2.068 (9)	C11—N2	1.387 (10)
Au1—C10	2.035 (8)	C11—C16	1.392 (10)
C1—N1	1.124 (11)	C11—C12	1.414 (10)
C2—C3	1.391 (10)	C12—C13	1.382 (12)
C2—C7	1.403 (10)	C12—C17	1.510 (11)
C2—N1	1.409 (9)	C13—C14	1.390 (12)
C3—C4	1.403 (13)	C13—H13	0.9400
C3—C8	1.517 (12)	C14—C15	1.375 (11)
C4—C5	1.353 (14)	C14—H14	0.9400
C4—H4	0.9400	C15—C16	1.390 (10)
C5—C6	1.381 (13)	C15—H15	0.9400
C5—H5	0.9400	C16—C18	1.507 (10)
C6—C7	1.391 (11)	C17—H17A	0.9700
C6—H6	0.9400	C17—H17B	0.9700
C7—C9	1.503 (10)	C17—H17C	0.9700
C8—H8A	0.9700	C18—H18A	0.9700
C8—H8B	0.9700	C18—H18B	0.9700
C8—H8C	0.9700	C18—H18C	0.9700
C9—H9A	0.9700	B1—F4	1.299 (14)
C9—H9B	0.9700	B1—F2	1.351 (11)
C9—H9C	0.9700	B1—F1	1.386 (11)
C10—N2	1.154 (11)	B1—F3	1.397 (15)
			
N1—C1—Au1	171.2 (7)	C13—C12—C17	121.9 (7)
C3—C2—C7	123.5 (7)	C11—C12—C17	121.3 (7)
C3—C2—N1	119.6 (7)	C12—C13—C14	120.6 (7)
C7—C2—N1	116.9 (6)	C12—C13—H13	119.7
C2—C3—C4	116.2 (7)	C14—C13—H13	119.7
C2—C3—C8	120.3 (8)	C15—C14—C13	121.0 (7)
C4—C3—C8	123.5 (8)	C15—C14—H14	119.5
C5—C4—C3	121.8 (7)	C13—C14—H14	119.5
C5—C4—H4	119.1	C14—C15—C16	121.2 (7)
C3—C4—H4	119.1	C14—C15—H15	119.4
C4—C5—C6	120.8 (7)	C16—C15—H15	119.4
C4—C5—H5	119.6	C15—C16—C11	116.7 (6)
C6—C5—H5	119.6	C15—C16—C18	121.6 (7)
C5—C6—C7	120.7 (8)	C11—C16—C18	121.7 (6)
C5—C6—H6	119.6	C12—C17—H17A	109.5
C7—C6—H6	119.6	C12—C17—H17B	109.5
C6—C7—C2	116.9 (6)	H17A—C17—H17B	109.5
C6—C7—C9	120.6 (7)	C12—C17—H17C	109.5
C2—C7—C9	122.4 (7)	H17A—C17—H17C	109.5
C3—C8—H8A	109.5	H17B—C17—H17C	109.5
C3—C8—H8B	109.5	C16—C18—H18A	109.5
H8A—C8—H8B	109.5	C16—C18—H18B	109.5
C3—C8—H8C	109.5	H18A—C18—H18B	109.5
H8A—C8—H8C	109.5	C16—C18—H18C	109.5
H8B—C8—H8C	109.5	H18A—C18—H18C	109.5
C7—C9—H9A	109.5	H18B—C18—H18C	109.5
C7—C9—H9B	109.5	F4—B1—F2	115.9 (12)
H9A—C9—H9B	109.5	F4—B1—F1	110.0 (8)
C7—C9—H9C	109.5	F2—B1—F1	111.8 (8)
H9A—C9—H9C	109.5	F4—B1—F3	109.4 (12)
H9B—C9—H9C	109.5	F2—B1—F3	102.4 (8)
N2—C10—Au1	171.3 (6)	F1—B1—F3	106.8 (9)
N2—C11—C16	117.5 (6)	C1—N1—C2	177.2 (7)
N2—C11—C12	118.8 (6)	C10—N2—C11	176.8 (7)
C16—C11—C12	123.7 (6)	C10—Au1—C1	173.7 (3)
C13—C12—C11	116.8 (7)		

## References

[bb1] Balch, A. & Parks, J. E. (1973). *J. Organomet. Chem.* **57**, C103–C106.

[bb2] Balch, A. & Parks, J. E. (1974). *J. Organomet. Chem.* **71**, 453–463.

[bb3] Bonati, F. & Minghetti, G. (1973). *Gazz. Chim. Ital.* **103**, 373–386.

[bb4] Bruker (2003). *SMART* and *SADABS* Bruker AXS Inc., Madison, Wisconsin, USA.

[bb5] Bruker (2006). *SAINT* Bruker AXS Inc., Madison, Wisconsin, USA.

[bb6] Burla, M. C., Caliandro, R., Camalli, M., Carrozzini, B., Cascarano, G. L., De Caro, L., Giacovazzo, C., Polidori, G. & Spagna, R. (2005). *J. Appl. Cryst* **38**, 381–388.

[bb7] Farrugia, L. J. (1997). *J. Appl. Cryst.* **30**, 565.

[bb8] Farrugia, L. J. (1999). *J. Appl. Cryst.* **32**, 837–838.

[bb9] Schmidbaur, H., Angermaier, K., Bauer, A., Sladek, A. & Schneider, W. (1997). *Z. Naturforsch.* **52**, 53–56.

[bb10] Schmidbaur, H., Ehlich, H. & Schier, A. (2002). *Z. Naturforsch. Teil B*, **57**, 890–894.

[bb11] Sheldrick, G. M. (2008). *Acta Cryst.* A**64**, 112–122. 10.1107/S0108767307043930 18156677

